# Exposing and Overcoming Limitations of Clinical Laboratory Tests in COVID-19 by Adding Immunological Parameters; A Retrospective Cohort Analysis and Pilot Study

**DOI:** 10.3389/fimmu.2022.902837

**Published:** 2022-06-29

**Authors:** Adrián Sánchez-Montalvá, Daniel Álvarez-Sierra, Mónica Martínez-Gallo, Janire Perurena-Prieto, Iria Arrese-Muñoz, Juan Carlos Ruiz-Rodríguez, Juan Espinosa-Pereiro, Pau Bosch-Nicolau, Xavier Martínez-Gómez, Andrés Antón, Ferran Martínez-Valle, Mar Riveiro-Barciela, Albert Blanco-Grau, Francisco Rodríguez-Frias, Pol Castellano-Escuder, Elisabet Poyatos-Canton, Jordi Bas-Minguet, Eva Martínez-Cáceres, Alex Sánchez-Pla, Coral Zurera-Egea, Aina Teniente-Serra, Manuel Hernández-González, Ricardo Pujol-Borrell, the “Hospital Vall d’Hebron Group for the study of COVID-19 immune profile”, Artur Llobell Uriel

**Affiliations:** ^1^ Infectious Disease Department, Hospital Universitari Vall Hebron, Barcelona, Spain; ^2^ International Health Program Institut Català de la Salut, Vall Hebron Research Institute (VHIR), Barcelona, Spain; ^3^ Department of Medicine, Universitat Autònoma Barcelona, Barcelona, Spain; ^4^ Centro de Investigación Biomédica en Red de Enfermedades Infecciosas (CIBERINFEC), Instituto de Salud Carlos III, Barcelona, Spain; ^5^ Translational Immunology Research Group, Vall Hebron Research Institute (VHIR), Barcelona, Spain; ^6^ Immunology Department, Hospital Universitari Vall Hebron, Barcelona, Spain; ^7^ Department of Cell Biology, Physiology, and Immunology, Universitat Autònoma Barcelona, Barcelona, Spain; ^8^ Intensive Medicine Department, Hospital Universitari Vall Hebron, Barcelona, Spain; ^9^ Organ Dysfunction and Resuscitation Research Group, Vall Hebron Research Institute (VHIR), Barcelona, Spain; ^10^ Epidemiology and Public Health Department, Hospital Universitari Vall Hebron, Barcelona, Spain; ^11^ Epidemiology and Public Health Research Group, Vall Hebron Research Institute (VHIR), Barcelona, Spain; ^12^ Department of Pediatrics, Obstetrics and Gynecology, Epidemiology and Public Health, Universitat Autònoma Barcelona, Barcelona, Spain; ^13^ Microbiology Department, Hospital Universitari Vall Hebron, Barcelona, Spain; ^14^ Microbiology Research Group, Vall Hebron Research Institute (VHIR), Barcelona, Spain; ^15^ Department of Genetics and Microbiology, Autonomous University of Barcelona, Barcelona, Spain; ^16^ Internal Medicine Department, Hospital Universitari Vall Hebron, Barcelona, Spain; ^17^ Systemic Disease Research Group, Valle Hebron Research Institute (VHIR), Barcelona, Spain; ^18^ Liver Disease Research Group, Valle Hebron Research Institute (VHIR), Barcelona, Spain; ^19^ CIBERehd - Instituto de Salud Carlos III, Barcelona, Spain; ^20^ Clinical Biochemistry Department, Hospital Universitari Vall d'Hebron and Clinical Biochemistry Research Group, Valle Hebron Research Institute (VHIR), Barcelona, Spain; ^21^ Bioinformatics and Statistics Group, University of Barcelona, Barcelona, Spain; ^22^ Immunology Division, Bellvitge University Hospital, Hospitalet de Llobregat, Barcelona, Spain; ^23^ Immunology Group, Germans Trias i Pujol Health Sciences Research Institute (IGTP), Badalona (Barcelona), Spain; ^24^ Immunology Department, Hospital Universitari Germans Trias Pujol, Badalona (Barcelona), Spain; ^25^ Statistics and Bioinformatics Unit, Vall Hebron Research Institute (VHIR), Barcelona, Spain

**Keywords:** SARS-CoV-2 infection, predictive risk-profile, clinical laboratory tests, cytokines, chemokines, acute phase reactants, CXCL10, flow cytometry

## Abstract

**Background:**

Two years since the onset of the COVID-19 pandemic no predictive algorithm has been generally adopted for clinical management and in most algorithms the contribution of laboratory variables is limited.

**Objectives:**

To measure the predictive performance of currently used clinical laboratory tests alone or combined with clinical variables and explore the predictive power of immunological tests adequate for clinical laboratories. Methods: Data from 2,600 COVID-19 patients of the first wave of the pandemic in the Barcelona area (exploratory cohort of 1,579, validation cohorts of 598 and 423 patients) including clinical parameters and laboratory tests were retrospectively collected. 28-day survival and maximal severity were the main outcomes considered in the multiparametric classical and machine learning statistical analysis. A pilot study was conducted in two subgroups (n=74 and n=41) measuring 17 cytokines and 27 lymphocyte phenotypes respectively.

**Findings:**

1) Despite a strong association of clinical and laboratory variables with the outcomes in classical pairwise analysis, the contribution of laboratory tests to the combined prediction power was limited by redundancy. Laboratory variables reflected only two types of processes: inflammation and organ damage but none reflected the immune response, one major determinant of prognosis. 2) Eight of the thirty variables: age, comorbidity index, oxygen saturation to fraction of inspired oxygen ratio, neutrophil-lymphocyte ratio, C-reactive protein, aspartate aminotransferase/alanine aminotransferase ratio, fibrinogen, and glomerular filtration rate captured most of the combined statistical predictive power. 3) The interpretation of clinical and laboratory variables was moderately improved by grouping them in two categories i.e., inflammation related biomarkers and organ damage related biomarkers; Age and organ damage-related biomarker tests were the best predictors of survival, and inflammatory-related ones were the best predictors of severity. 4) The pilot study identified immunological tests (CXCL10, IL-6, IL-1RA and CCL2), that performed better than most currently used laboratory tests.

**Conclusions:**

Laboratory tests for clinical management of COVID 19 patients are valuable but limited predictors due to redundancy; this limitation could be overcome by adding immunological tests with independent predictive power. Understanding the limitations of tests in use would improve their interpretation and simplify clinical management but a systematic search for better immunological biomarkers is urgent and feasible.

## Introduction

Over two years after the onset of the coronavirus disease (COVID-19) pandemic, the clinical, laboratory, and imaging features of patients with severe acute respiratory syndrome coronavirus 2 (SARS-CoV-2) infection have been widely described (1–4). The wide clinical spectrum of COVID-19 became obvious during the first wave, and although the effect of inoculum size should be considered (5, 6), variation has been mainly attributed to host factors, as variants of concern only appeared later (7) (8). The analysis of the first wave has therefore obvious advantages for the identification of host factors and their biomarkers. Among host factors that affect the severity of illness, age, sex, genetic background, immunological status and prior immunity to coronaviruses (9) have been evaluated. Gene mutations of the type interferon (IFN) pathway (10) and antibodies to type IFNs play a clear role in a small proportion of cases (11); polymorphisms in several genes associated with immune response have been identified in genome-wide association studies (12, 13); however, to date, the genotypes that convey a risk of severe COVID-19 have not been defined in a way that is practically applicable for prediction in clinical practice.

Reports originating from the analysis of electronic health records have confirmed the predictive value of clinical laboratory tests usually associated with poor outcomes in other infections i.e., blood cell counts, acute-phase reactants (APRs), and coagulation factors (14–22) but none of the proposed predictive algorithms combining demographic, clinical, and laboratory data have been widely adopted. In small case series, the state of the immune system in COVID-19 patients has been analyzed using the latest tools (23–30) leading to the detection of deep perturbations in the immune system. However, inferences of the effect of these perturbations in the efficiency of the immune response and their clinical consequences are not simple and, to date, no new predictive tests have been validated and added to the clinical laboratory toolbox for COVID-19 management, reflecting not only intrinsic technical difficulties, but also the excessive separation between research and clinical laboratories.

We report a retrospective analysis of data from a cohort of 1,579 consecutive patients treated at the Vall d’Hebron University Hospital (HUVH) during the first wave of COVID-19 in Barcelona. We validated the main conclusions by comparison with cohorts from two other academic hospitals that belong to the same healthcare provider (the Catalan Institute of Health [ICS]) in Catalonia, Spain.

We initiated the study with the hypothesis that the predictive power of clinical laboratory tests had not been fully exploited and with the main objective of improving their interpretation. A secondary objective was to explore a selection of robust immunological tests that might identify an early dysregulated immune response associated with severe COVID-19, with the hypothesis that these tests could provide additional non-redundant prediction power.

## Patients and Methods

### Patients

**Figure 1:**
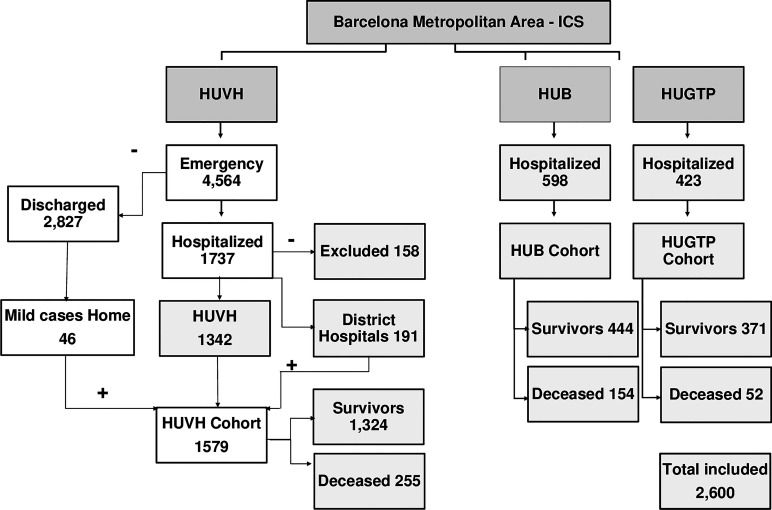
Selection of patients for the cohorts from Vall d’Hebron University Hospital (HUVH), Bellvitge University Hospital (HUB), and Germans Trias i Pujol University Hospital (HUGTP). All patients were confirmed by polymerase chain reaction (PCR) to have coronavirus disease (COVID-19). The details of the excluded patients are provided in **Table S1**. The data from HUVH corresponds to patients who were admitted to the emergency division between 10 March and 29 April 2020; to HUGTP between 17 March and 12 May 2020; and to HUB between 16 March and 23 September 2020. The number of deceased patients corresponds to the 28-day follow-up period. The HUB and HUGTP cohorts include only hospitalised patients but in the HUVH cohort, 46 patients were discharged home or to a medicalized hotel within 24h and monitored by the primary care network.

The database of the HUVH cohort was obtained by merging data sets from the Infectious Disease, Epidemiology and Public Health, and Clinical Laboratory departments. Consecutive patients aged ≥18 years with a SARS-CoV-2 positive polymerase chain reaction (PCR) from any respiratory sample, hospitalized in HUVH between 10 March and 29 April 2020 were included in the study (see **Tables 1**, **S1**). This COVID-19 HUVH cohort consisted of 1,579 patients (**Figure 1**). All patient medical records included the main symptoms, days from symptom onset (DFSO), initial assessment of vital signs, comorbidities, length of hospital stay (LOS), intensive care unit (ICU) admission, oxygen supplementation and supportive ventilation requirements, outcome during the hospitalization and results from clinical laboratory tests. Data were censored on the date of discharge, death, or 28 days after admission, whichever occurred first. The outcome of all patients discharged before the 28th day was ascertained through a review of the primary care electronic health record annotations.

**Table 1:** Summary of the clinical and demographic features of HUVH cohort vs outcome.

		Survivors vs deceased				Non-severe vs severe		
		Patients n (%)	Survivors n (%)	Deceased n (%)	*p*-value	Non-severe n (%)	Severe n (%)	*p*-value
**All**		1,579 (100)	1324 (83.9)	255 (16.1)		1040 (63.8)	539 (34.1)	
**Female**		699 (44.3)	592 (44.7)	107 (42.0)	0.449	491 (47.2)	208 (38.6)	**0.001**
**Male**		880 (55.7)	732 (55.3)	148 (58.0)		549 (52.8)	331 (61.4)	
**ICU**		236 (14.9)	203 (15.3)	33 (13.9)	0.4	1 (0.1)	235 (43.6)	**1.612E-126**
		Median (IQR)	Median (IQR)	Median (IQR)		Median (IQR)	Median (IQR)	
**Age, years**		62 (50–75)	57 (48–70)	82 (74–87)	**7.26E-81**	58 (47–71)	70 (54–82)	**2.4872E-29**
**DFSO**		7 (4–9)	7 (5–10)	5 (2–7)	**9.40E-19**	7 (5–10)	6 (3–8)	**2.9561E-14**
**LOS**		7 (2–20)	7 (2–24)	7 (4–11)	0.4226	5 (2–10)	14 (6–36)	**1.5748E-44**
**Disease duration, days**		15.0 (10–28)	15 (10–34)	12 (9–18)	**4.45E-11**	13 (9–21)	22 (11–43)	**1.4952E-17**
**Clinical Presentation**		n (%)	n (%)	n (%)		n (%)	n (%)	
**General**	Fever	1314 (83.2)	1132 (85.4)	199 (75.3)	0.040	886 (85.2)	438 (81.3)	0.051
**Respiratory**	Upper respiratory symptoms (only)	94 (5.9)	85 (6.5)	9 (3.4)	0.063	69 (6.5)	25 (4.6)	0.118
	Lower respiratory symptoms	1351 (85.6)	1148 (86.7)	203 (79.6)	0.020	892 (85.8)	459 (85.2)	0.763
	Pneumonia	1524 (96.5)	1271 (96.0)	253 (99.2)	0.008	989 (95.1)	535 (99.3)	**3.62E-06**
**Digestive**	All	491 (31.1)	440 (33.2)	51 (20.0)	1.182E-05	353 (33.9)	138 (25.6)	**7.11E-04**
**Comorbidities**		n (%)	n (%)	n (%)		n (%)	n (%)	
	Cardiovascular & hypertension	712 (45.1)	508 (38.4)	204 (80.0)	**8.534E-37**	394 (37.9)	318 (59.0)	**1.463E-15**
	Chronic lung disease	278 (17.6)	198 (15.0)	80 (31.4)	**5.134E-09**	145 (13.9)	133 (24.7)	**2.143E-07**
	Diabetes	293 (1850.0)	223 (16.8)	70 (28.3)	**1.90E-05**	170 (16.3)	123 (22.8)	**0.002**
	Neurological disease	227 (14.4)	156 (11.8)	71 (27.8)	**1.29E-09**	121 (11.6)	106 (19.7)	**2.89E-05**
	Chronic kidney disease	134 (8.5)	79 (6)	55 (21.6)	**1.03E-13**	55 (5.3)	79 (14.7)	**9.101E-10**
	Active non-terminal malignancy	113 (7.2)	62 (4.7)	51 (20.0)	**1.10E-15**	41 (3.9)	72 (13.4)	**4.197E-11**
	Obesity	261 (16.5)	227 (17.3)	34 (12.8)	0.85	145 (13.9)	116 (21.5)	**2.00E-04**
	Chronic liver disease	61 (3.9)	48 (3.7)	13 (4.9)	0.139	37 (3.6)	24 (4.5)	0.409
Comorbidity index*								
		1 (0-2)	2 (1-3)	2 (1-3)	**2.2961E-38**	1 (0-2)	2 (1-3)	**1.0118E-26**

*The breakdown of patients by comorbidity index is in **Table S4**. Exact p-values from the Mann–Whitney U test, Significancy should be considered for p ≤ 0.001 after Bonferroni. F, female; M, male; DFSO, days from symptom onset; LOS, length of stay; significant p values in bold.

Comorbidities were classified as 1) cardiovascular disease and/or hypertension, 2) chronic lung disease, 3) diabetes, 4) neurological disease, 5) chronic kidney disease, 6) active non-terminal malignancy, 7) obesity, and 8) chronic liver disease. Each comorbidity was assigned value of 1, and a global comorbidity index (1 to 8) was generated. The clinical severity category was assigned as the maximal score attained during hospitalization, using a simplified version of the World Health Organization (WHO) 10-point COVID-19 disease clinical progression score (31) as follows: 1) Mild, no activity limitations or not requiring hospitalization; 2) Moderate, hospitalized, not requiring high-flow oxygen therapy or ventilation support; 3) Severe, hospitalized requiring high-flow oxygen therapy or ventilation support; and 4) Deceased, those who died before day 28 of hospitalization. These categories correspond to the WHO scores 1–3, 4–5, 6–9, and 10, respectively. For some analyses, the mild and moderate categories were combined into a non-severe category, and the severe and deceased categories were combined into a severe category.

The validation cohorts from the Bellvitge University Hospital (HUB) and the Germans Trias i Pujol University Hospital (HUGTP) included 598 and 423 patients, respectively, and, together with the HUVH cohort, at total of 2,600 patients were included in the analysis (**Figure 1**).

### Outcomes

Final outcomes for comparison included survival vs. death, and maximum clinical severity. For the validation cohorts the only available outcome was survival for 28 days (survivors) and death (deceased).

### Clinical Laboratory Tests

Detection of SARS-CoV-2 was first performed by an in-house PCR assay with primers and probes from 2019-nCoV CDC PCR panel, using the One-Step RT-PCR kit (Qiagen, Germany). When commercial assays became available, a real-time multiplex RT-PCR assay (Laplet 2019-nCoV Assay, Seegene, South Korea) was used.

The clinical laboratories were equipped with Beckman Coulter (Brea, CA, USA) and Roche Diagnostics (Indianapolis, IN, USA) automatic analyzers that were integrated with two TECAN (Zug, Switzerland) continuous lines and two automatic cold storage and retrieval units that ensure sample integrity. IL-6 levels were measured in a Elycsis^®^ Cobas analyzer (Roche). Samples for assessing the predictive performance of clinical laboratory tests were taken on admission to the hospital; glomerular filtration rate (GFR) was calculated by applying the algorithm of Levey et al. (32); additional laboratory test data for the 28-day follow-up period were available from 9,475 samples corresponding to 1,079 of the 1,579 patients in the HUVH cohort.

### Immunological Tests

The levels of CCL2, CXCL10, GM-CSF, IFN-alpha, IFN-gamma, IL-10, IL-12 p70, IL-13, IL-15, IL-17A, IL-1RA, IL-2, IL-4, IL-6, IL-7, TNF and granzyme B were measured in sera using the ELLA microfluidic platform (Biotechne^®^, Minneapolis, MN, USA); sCD163 levels were measured by a commercial ELISA (CD163 human kit, Thermo Fisher Societies, Waltham, MA, USA).

The Human Immune Phenotyping Consortium protocol (33, 34) was adapted for the study of COVID-19 patients. The antibodies used are shown in **Table S2**. Blood was collected in EDTA vacutainer tubes (BD-Plymouth, UK) and processed within 4 hours. Lymphocytes were selected by CD45 and SSC including 10^5^ cells in the gate. In samples with marked lymphopenia a lower number were selected. Cells were analyzed in a NAVIOS EX flow cytometer (Beckman Coulter). Data were analyzed with Kaluza Beckman Software v.2.1. Absolute values were generated by loading counts from the hematological analyzer (XN-2000; Sysmex, Japan) parallel sample analysis.

### Statistical Analysis

Categorical variables were summarized as frequencies and proportions and continuous variables as means, standard deviations, and 95% confidence intervals (CIs) or medians and interquartile ranges (IQRs), depending on their distribution. Pairwise comparisons used the Mann–Whitney U-test and Kruskal-Wallis test, adjusted for the false-discovery rate (FDR) using the Benjamini and Hochberg, or corrected by the Bonferroni method where indicated. C-reactive protein (CRP), IL-6, ferritin, and D-dimer values were logarithmically transformed. A threshold of 30% of laboratory missing data was used as the exclusion criteria for data analyses. The initial oxygen saturation to fraction of inspired oxygen ratio (SpO2/FiO2) was available for a subset of 827 patients. The data from these patients were either analyzed separately, or when this parameter was included in a general analysis, this was indicated. Data from treatments were available in 981 patients with comparable clinical and demographic features as patients in severity categories moderate, severe, and deceased of the HUVH cohort (**Table S3**).

Bivariable logistic regression was used to calculate the age-adjusted odd ratios (OR) and effect size (Z score) of each variable. Multivariable logistic regression was used to calculate the predictive power of different combinations of variables. Correlation among variables was analyzed using the non-parametric Spearman test. For analysis of follow-up data of the HUVH cohort, locally weighted smoothing (LOESS) was applied to clinical laboratory variables to visualize the relationship between the mean and CI of each variable, time and 28-day outcome, as described in 4. To assess the performance of each clinical laboratory test, the receiver-operating characteristic (ROC) curve and the corresponding area under the curve (AUC) values were calculated, using age as a variable for comparison. In addition, random forest simulation, as machine learning method, and principal component analysis (PCA) were performed to further compare the influence of the laboratory and clinical variables on the outcomes in each hospital dataset.

Statistical tests were 2-sided and used a significance threshold of at least p <0.05. R, version 4.1.0 (The R Foundation for Statistical Computing, Vienna, Austria) and Prism 9^®^ (GraphPad, San Diego, CA, USA) packages were used for all analyses. Statistical analysis was conducted by the Statistics and Bioinformatics Unit (UEB), Vall d’Hebron Hospital Research Institute, and by co-authors PC-E and RP-B under the supervision of the UEB.

## Results

### Overall Clinical Features of HUVH Cohort

**Figure 2:**
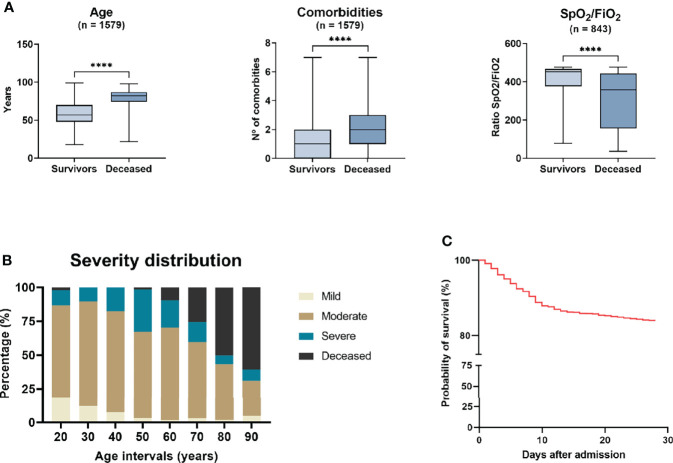
The structure and outcomes of the Vall d’Hebron University Hospital cohort. **(A)** Left panel, age distribution of the survivors and that of the deceased is markedly different (median [IQR]: 62 years [50–75 years] vs. 82 years [74–87 years], p<0.001) as are comorbidities (central panel) and SpO2/FiO2 (right panel). **(B)** Distribution of the patients in the HUVH cohort among the four severity categories, based on the World Health Organization criteria (described in the Material and Methods section). The number of patients in the mild category is small (n=71) as only patients with bilateral pneumonia or severe associated pathologies were hospitalised during this period of the pandemic. **(C)** Survival after admission: this graph highlights mortality during the initial 10 days, with a high number of patients older than 80 years dying in the initial 3–4 days (see text “Overall Clinical Features of HUVH cohort”). HUVH, Vall d’Hebron University Hospital ****p < 0.0001.

The HUVH cohort included 1,579 PCR-confirmed COVID-19 patients with a median age of 62 years (IQR: 50–75 years), of whom 255 (16.1%) died during the first 28 days after hospitalization (**Figure 2A**). Eight hundred eighty (55.7%) patients were male. The proportion of males was higher than females (58.0%) among the deceased patients and this proportion of males was significantly higher than their proportion in the Barcelona metropolitan area at the time (47.5% male, p <0.001, (35). A total of 236 (14.9%) patients were admitted to the ICU with a 28-day case fatality rate of 13.9% for this subgroup (36).

The presenting symptoms are shown in **Table 1**. Cardiovascular and/or hypertension, chronic lung disease, diabetes, neurological disease, chronic kidney disease, and active non-terminal malignancy were significantly associated to decreased 28-day survival, but not chronic liver disease nor obesity. Of note, digestive symptoms were more frequent in survivors (31.1% vs. 20.0%, p <0.001). The comorbidity index was significantly higher in deceased patients and patients with severe disease than in survivors and patients with non-severe disease. Each comorbidity added 10% mortality risk up to an index of 4 (**Table S4**).

The distribution of disease severity was as follows, 71 (4.5%), 969 (61.4%), 284 (17.9%), and 255 (16.1%) in the mild, moderate, severe, and deceased categories, respectively. Among the mild patients, 46 were discharged within 24h. The age of patients increased with disease severity category, except between the moderate and severe disease groups (**Figure 2B** and **Table S4**). The LOS increased with disease severity for the three initial disease severity categories but was shorter among the deceased because 24.9% of obits occurred during the initial 4 days of hospitalization (**Figure 2C**). The median disease duration was 18 days (IQR: 10–18 days) and was progressively longer with increasing disease severity. Age had a strong effect on mortality: for patients in the age groups 50–59, 60–69, 70–79, 80–89 and >90 years, with 28-day case fatality rates of 1.82%, 10.9%, 26.4%, 49.7% and 60.6% respectively (**Table S5**).

The treatment was available in 981cases in the exploratory cohort and can be consulted in **Table S3**. Most patients received hydroxychloroquine (90.5%) and antivirals (87.7%) following the recommendations of treatments at that stage, but a proportion also received the drugs that were later found to be effective such corticosteroids (18.3%) and Tocilizumab (25.1%).

In the dichotomous disease severity grouping, there were 1,040 and 539 patients in the non-severe and severe categories, respectively. Deceased patients accounted for 43.7% of the severe category. The disease severity was significantly associated with age, DFSO, LOS, disease duration, and comorbidities other than chronic liver disease (*p*=2.4·10^-29^, *p*=2,9·10^-14^, *p*=1.5·10^-44^, p=1.4·10^-17^ respectively). Disease severity was greater in males than in females, but after adjusting for multiple comparisons the statistical significance was moderate compared with the other significant associations (exact p =0.001, after Bonferroni’s correction p=0.03) (**Table 1**).

### Predictive Power of Current Clinical Laboratory Tests

**Figure 3:**
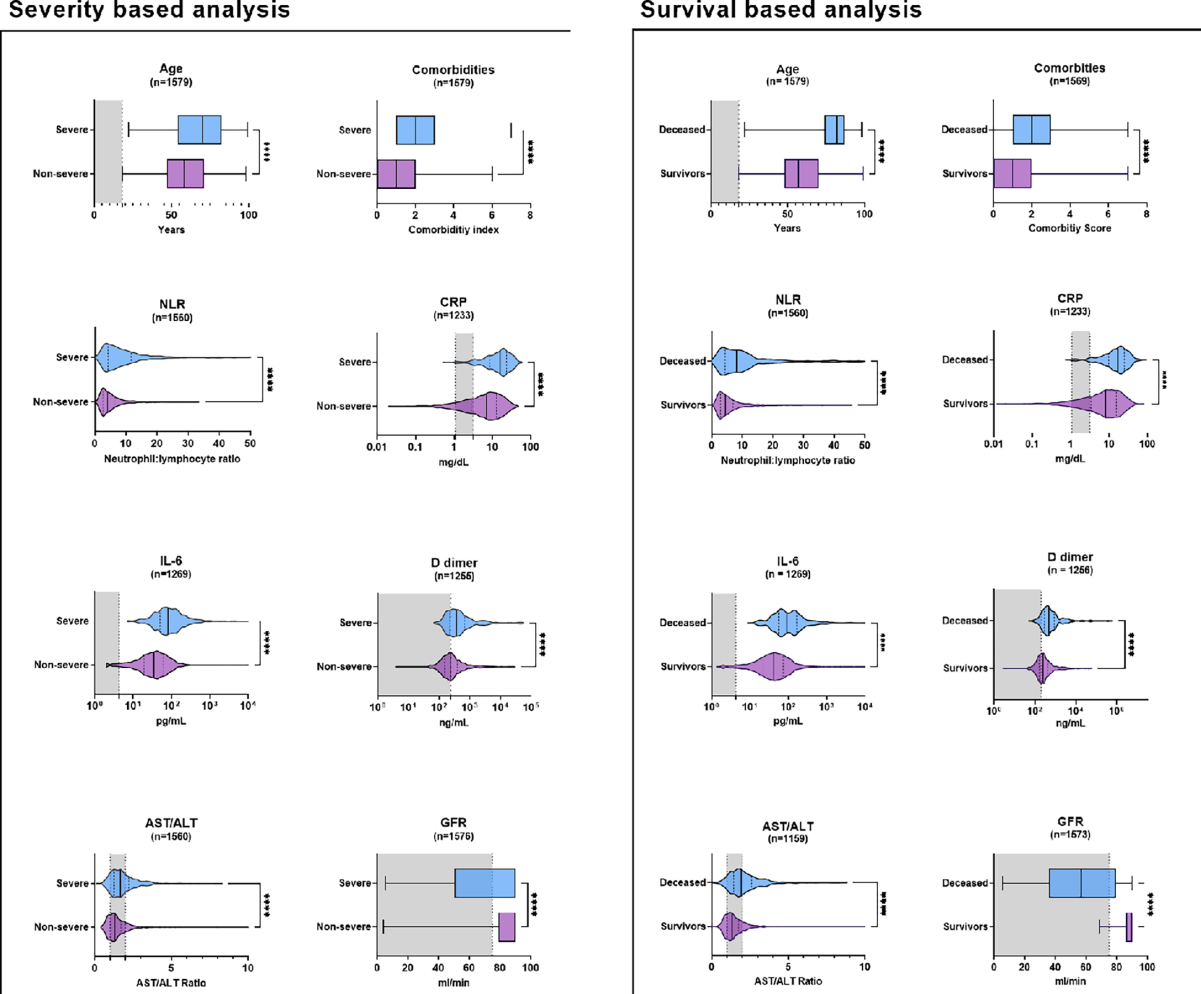
Univariate comparisons of a selection of clinical laboratory-derived variables at admission and 28-days survival for the survival/decease and non-severe, severe outcomes in the Vall d’Hebron University Hospital cohort. n, number of cases plotted; NLR, neutrophil-to-lymphocyte ratio; CRP, C-reactive protein; AST, aspartate aminotransferase; ALT, alanine aminotransferase; GFR, glomerular filtration rate. ****p < 0.0001. When non-significant, the numerical p-values are given. The exact p-values are given in **Table 2**. The distribution of age and GFR are markedly different in both the severity and survival analysis.

The exploratory statistical analysis of the HUVH cohort revealed strong association of 22 of the 30 variables with 28-day outcomes (**Figure 3** and **Tables 2** and **S6**). However, the analysis of the classification tables generated by iterative logistic regression analysis using different sets of variables showed that, despite good ROC curves (see below), their power in predicting poor outcomes, either decease or severe disease, was under the 60% (**Table 3** and **Supplementary Tables** in excel format “Repeated Multiple Logistic Regression”). Of concern, prediction was very dependent on age (**Table 3**). Laboratory variables by themselves -without SpO2/FiO2- are poor predictors specially of decease (34.78% of correctly classified patients). Analyses with a reduced set of eight variables i.e., age, comorbidities, SpO2/FiO2, NLR, CRP, AST/ALT, fibrinogen and GFR, gave similar results as those using all variables, confirming the redundancy of many variables. Machine learning analysis using random forest confirmed this low prediction power of laboratory variables on their own. See below "Selection of a core panel of clinical laboratory tests".

**Table 2:** Pairwise comparison of biomarkers for decease and severity outcomes, HUVH cohort.

Patients,	Total (n=1,579)	Survivors (n=1,324)	Non-survivors (n=255)	*p*-value (exact)	Non-Severe (n = 1040)	Severe and deceased (n = 539)	*p*-value (exact)
Age, years, median and IQR	62 (50–75)	57 (48–70)	82 (74–87)	7.26E-81	58 (47-71)	70 (54-82)	2.49E-29
Comorbidities	1 (0-2)	2 (1-3)	2 (1-3)	2.30E-38	1 (0-2)	2 (1-3)	1.0118E-26
**INFLAMMATION RELATED BIOMARKERS (IFRB)**							
**Blood (normal range)**							
Hb (12–15 g/dL)	13.5 (12.3–14.5)	13.7 (10.7–11.9)	12.6 (11.4–13.8)	4.95E-15	13.7 (12.5-14.6)	13.1 (12.0-14.3)	5.64E-09
WBC (4–11 10^9^/L)	6.5 (5.0–8.8)	6.5 (5.0–8.2)	7.5 (5.32–10)	1.51E-05	6.36 (4.95-8.10)	7.39 (5.29-10.4)	1.26E-10
Neutrophils, % (40–80)	76.1 (68–83.2)	74.8 (68.3–81.0)	82.9 (75.4–87.9)	1.07E-20	73.3 (65.9-79.8)	82.4 (74.3-87.6)	6.07E-39
Neutrophils (2–7 109/L)	4.8 (3.5–6.7)	4.7 (3.4–7.6)	6.1 (3.9–8.6)	7.18E-10	4.56 (3.33-6.23)	5.92 (4.01-8.68)	1.63E-19
Lymphocytes, % (20–50)	16 (10.5–23)	17 (12–24)	10 (7.2–17.0)	1.07E-23	18.1 (12.8-24.8)	11.6 (7.50-18.2)	8.34E-41
Lymphocytes (1.2–3.5 10^9^/L)	1 (0.7–1.4)	1.1 (0.8–1.4)	0.7 (0.6–1.0)	3.69E-22	1.11 (0.82-1.50)	0.85 (0.60)	1.80E-28
Monocytes, % (2–10)	6.7 (4.8–8.8)	6.8 (5.0–8.8)	5.4 (3.6–7.80)	1.01E-10	7.20 (5.50-9-20)	5.40 (3.70-7.80)	1.92E-24
Monocytes (0.1–1 10^9^/L)	0.43 (0.30–0.59)	0.44 (0.31–0.59)	0.39 (0.28–0.56)	0.013	0.45 (0.33-0.61)	0.39 (0.28-0.58)	1.02E-05
Eosinophils, % (0.0–5.0)	0.0 (0–0.3)	0.00 (0.00–0.10)	0 (0.00–0.00)	4.40E-07	0.1 (0.00-0.40)	0.00 (0.00-0.10)	1.87E-14
Eosinophils (0.0–0.5 10^9^/L)	0.00 (0.00–0.01)	0.0 (0–0.01)	0 (0.00–0.00)	0.006	0.0 (0–0.03)	0 (0.00–0.01)	9.71E-12
Basophils (0.0–0.2 E9/L)	0 (0.01–0.03)	0.0 (0.01–0.03)	0.02 (0.01–0.03)	0.454	0.02 (0.01–0.02)	0.02 (0.01–0.03)	0.1591
Neutrophil–Lymphocyte ratio	4.8 (3.0–7.0)	4.4 (3.2–6.7)	7.7 (4.3–12.2)	1.39E-21	4.07 (2.68-4.21)	7.07 (4.21-11.7)	4.71E-39
Platelets (140–400 10^9^/L)	197 (154–251)	202 (170–286)	174 (133–227)	2.87E-07	292 (159-254)	190 (143-190)	2.63E-04
APR and related parameters							
CRP (0.03–0.5 mg/dL)	8.9 (3.8–16.6)	8.1 (3.8–15.5)	17.9 (10.2–24.5)	2.05E-19	7.07 (2.57-12.58)	15.8 (8.51-23.44)	5.99E-43
IL-6 (0.0–4.3 pg/mL)	45.1 (23.6–80.0)	41.41 (24.7–85.9)	90.5 (55.3–162.8)	1.88E-24	34.6 (19.0-61.65)	81.2 (50.1-138.0)	1.01E-55
Ferritin (25–400 ng/mL)	539 (282.5–1011.5)	527 (155.5–709.5)	671 (355–1153)	0.04	467 (251.-891.2)	724 (426.-1348.)	1.06E-12
Coagulation							
Fibrinogen (2.39–6.1 g/L)	5.1 (4.4–6)	5.2 (4.4–5.7)	4.9 (4.2–5.9)	0.04	5.06 (4.42-5.86)	5.31 (4.45-6.15)	0.0129
D-dimer (0–243 ng/mL)	263 (168–463.5)	248.5 (197.8–591.0)	477 (292.5–860.8)	4.65E-16	234 (151-389)	371 (223-692)	3.80E-20
Prothrombin time, INR (0.7–1.3)	1.1 (1.0–1.2)	1.1 (1.0–1.2)	1.1 (1.1–1.3)	9.32E-08	1.09 (1.02-1.17)	1.11 (1.04-1.23)	1.25E-05
**ORGAN DAMAGE RELATED BIOMARKERS (ODRB)**							
SpO2/FiO2	448 (354-462)	452 (377-465)	358 (156-443)	5.55E-22	457 (438-467)	369 (230-448)	8.58E-44
Liver function tests							
AST (12–50 IU/L)	40 (30–60)	39 (26.0–49.5)	44.5 (31–68)	0.01	38 (28-55)	45 (33-68)	6.55E-11
AST/ALT (<1.5)	1.39 (1.06-1.88)	1.3 (1.0-1.8)	1.9 (1.4-2.6)	6.06E-31	1.3 (1-1.71)	1.67 (1.24-2.21)	2.97E-24
Bilirubin, Direct (0.1–0.57 mg/dL)	0.30 (0.24–0.38)	0.30 (0.24–0.37)	0.35 (0.27–0.46)	8.37E-05	0.29 (0.24-0.36)	0.32 (0.26-0.43)	1.34E-05
Bilirubin, Total (0.3–1.2 mg/dL)	0.57 (0.45–0.74)	0.57 (0.44–0.73)	0.63 (0.47–0.85)	0.03	0.56 (0.45-0.73)	0.58 (0.44-0.81)	0.185
Kidney function tests							
Urea (17–42 mg/dL)	35 (25–51)	32 (24.0–55.0)	58 (42–87)	8.77E-47	31 (24-44)	46 (31.8-72.3)	2.23E-31
Creatinine (0,67 - 1.17 mg/dL)	0.80 (0.60–0.97)	0.79 (0.65–0.94)	1.01 (0.78–1.36)	2.20E-20	0.79 (0.64-0.95)	0.92 (0.74-1.29)	2.48E-23
GFR (>75 mL/min/1,73m^2^)	88.5 (67.8–90.0)	90.0 (76.4–90.0)	56.9 (35.9–79.5)	2.37E-101	90 (78.9-90)	76.3 (50.3-90)	1.42E-37

AST, Aspartic Amino Transferase; ALT, Alanine Amino Transferase; CRP; C Reactive Protein; GFR, Glomerular Filtration Rate; Hb, Hemoglobin; NLR, Neutrophil Lymphocyte Ratio; SpO2/FiO2, Oxygen saturation to fraction of inspired oxygen ratio; WBC, White Blood cell Count.

**Table 3:** Classification tables from multiple logistic regression including different sets of variables for survival vs decease or severe vs non-severe as outcome.

Predicted outcome, 19 clinical and laboratory variables* for survival versus decease				
Classification table	Predicted survival	Predicted decease	Total	% Correctly classified
Observed Survival	357	8	365	97.81
Observed Decease	21	25	46	54.35
Total	378	33	411	92.94
Predicted outcome, 19 clinical and laboratory variables* severe versus non-severe				
Classification table	Predicted non-severe	Predicted severe	Total	% Correctly classified
Observed non-severe	244	26	270	90.37
Observed severe	64	77	141	54.61
Total	308	103	411	78.10
Predicted outcome, 16 non-clinical variables** for survival versus decease				
Classification table	Predicted survival	Predicted decease	Total	% Correctly classified
Observed survival	356	9	365	97.53
Observed decease	30	16	46	34.78
Total	386	25	411	90.51
Predicted outcome, 16 non-clinical variables** for severe versus non-severe				
Classification table	Predicted non-severe	Predicted severe	Total	% Correctly classified
Observed non-severe	245	25	270	90.74
Observed severe	59	82	141	58.16
Total	304	107	411	79.56
Predicted outcome, 15 non-clinical variables for survival versus decease (no SpO2/FiO2).***				
Classification table	Predicted survival	Predicted decease	Total	% Correctly classified
Observed survival	897	9	906	99.01
Observed decease	68	20	88	22.73
Total	965	29	994	92.25
Predicted outcome, 15 non-clinical variables for severe versus non-severe (no SpO2/FiO2)***				
Classification table	Predicted non-severe	Predicted severe	Total	% Correctly classified
Observed non-severe	691	49	740	93.38
Observed severe	162	92	254	36.22
Total	853	141	994	78.77

*The 19 variables included were age, gender, comorbidity index, SpO2/FiO2, Hb, Neutrophil %, Lymph %, monocyte %, eosinophils %, NLR, Platelets, CRP, IL-6, D-dímer, ferritin, fibrinogen, prothrombin time INR, AST/ALT ratio and GFR. ** The 16 variables included were the same minus for age, sex, and comorbidities. *** The 15 variables were the same minus for age, sex, comorbidities and, SpO2/FiO2.

### Improving Interpretation of Current Clinical Laboratory Tests

The white blood cell differential counts showed marked imbalance due to an approximately 250% reduction in the lymphocyte count and a 20–30% increase in the neutrophil count. At the individual level, the reduction of lymphocytes was disproportionate to the increase in neutrophils.

The Acute Phase Reactants had a broad range of variation e.g., >10,000 and 50-fold for IL-6 and CRP, respectively, and in most patients the values were out of the normal range, while the aspartate aminotransferase/alanine aminotransferase (AST/ALT) ratio and kidney function test results were only moderately altered and often remained within the normal range.

**Figure 4:**
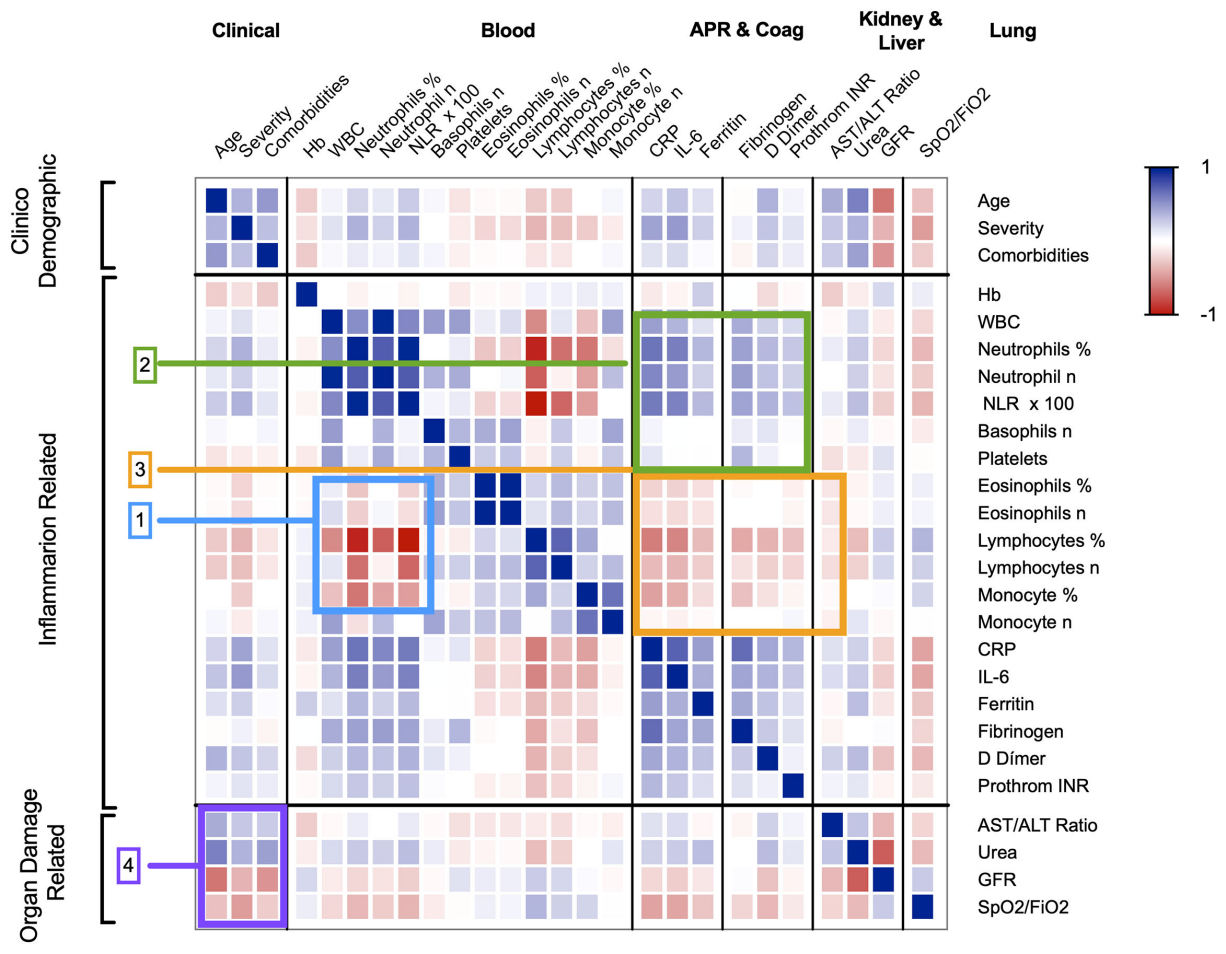
Overall correlograms of selected data on demographics and clinical laboratory variables that were organised in categories. [1] The blue rectangle highlights the negative correlation between neutrophils and the cluster of lymphocytes, monocytes, and eosinophils. [2] The green rectangle highlights the blood cell variables that correlate positively with the acute-phase reactants (APRs) and coagulation factors. [3] The orange rectangle highlights the negative correlation between lymphocytes, monocytes, and eosinophils with APRs and coagulation factors. [4] The magenta rectangle highlights the correlations between age, disease severity, comorbidities with liver and kidney function and SpO_2_/FiO_2_. The cells following the diagonal highlights the seven families of variables: clinical, blood cells, APR-coagulation, liver, kidney and lung tests, which show the expected strong correlations among themselves. The thick lines between rows separate the main categories. APR, acute-phase reactants; SpO_2_/FiO_2_, oxygen saturation/fraction of inspired oxygen; NLR, neutrophil-to-lymphocyte ratio; CRP, C-reactive protein; AST, aspartate aminotransferase; ALT, alanine aminotransferase; GFR, estimated glomerular filtration rate. The r- and p-values of the data represented in the heatmap are in xlsx format files in the supplementary material “Correlation of variables, r-values” and “Correlation of variables, p-values”.

Multiple correlation (**Figure 4**), multivariable logistic regression analyses (**Table 3**), age-adjusted logistic regression (**Table 4**), and examining their respective shifts from the normal range (**Table S7**), suggested that these variables could be classified into three broad categories, clinic-demographic (CD), including age, sex and the comorbidity index; inflammation related biomarkers (IFRB) including blood cell counts, levels of APRs, and coagulation factors; and organ damage-related biomarkers (ODRB), including liver and kidney function tests and SpO2/FiO2. These analyses also revealed that the neutrophil-lymphocyte ratio (NLR) and the AST/ALT ratio captured most of the predictive value of lymphocyte and neutrophils variations and of liver function test variations, respectively, and that SpO2/FiO2 conveyed much of the predictive power of the ODRBs (see supplementary text “Sequence of statistical biomarker analysis”).

**Table 4:** Bivariate age adjusted logistic regression for 28-days survival/decease and non-severe/severe outcomes.

28d decease outcome age adjusted						28 maximal severity age adjusted				
Variables	OR	CI	Z value	Pr(>|z|)	Z value Rank	OR	CI	Z value	Pr(>|z|)	Z value Rank
IL-6 (pg/mL)	2.59	2.06-3.31	7.84	<0.001	1	12.24	8.24-18.56	12.10	<0.001	1
CRP (mg/dL)	3.20	2.38-4.41	7.40	<0.001	2	9.55	6.47-14.40	11.06	<0.001	2
SpO2/FiO2 ratio	0.99	0.99-1.00	7.05	<0.001	3	0.99	0.98-0.99	10.17	0.001	4
Neutrophils (%)	1.06	1.04-1.08	6.38	<0.001	4	1.19	1.14-1.23	10.76	<0.001	3
NLR x 100	1.08	1.06-1.11	6.33	<0.001	5	1.13	1.10–1.16	9.87	0.001	5
Monocytes (%)	0.86	0.81-0.90	6.20	<0.001	6	0.86	0.83-0.89	7.89	<0.001	8
Neutrophils (10^9^/L)	1.14	1.09-1.19	5.74	<0.001	7	1.19	1.14-1.23	8.86	<0.001	7
GFR (mL/1.73 m^2^)	0.98	0.97-0.99	5.44	<0.001	8	0.98	0.97-0.98	6.94	<0.001	11
Lymphocytes (%)	0.95	0.92-0.97	5.04	<0.001	9	1.03	1.03-1.04	9.79	<0.001	6
WBC (10^9^/L)	1.11	1.06-1.16	4.82	<0.001	10	1.14	1.01-1.18	7.29	<0.001	9
AST/ALT ratio	1.49	1.26-176	4.74	<0.001	11	1.53	1.32-1.80	5.47	<0.002	13
D dimer (ng/mL)	1.51	1.26-1.83	4.35	<0.001	12	2.60	1.92-3.56	6.05	<0.001	12
Creatinine (mg/dL)	1.32	1.14-1.53	3.68	<0.001	13	1.39	1.20-1.65	4.02	<0.001	16
Lymphocytes (10^9^/L)	0.51	0.35-0.73	3.61	<0.001	14	0.41	0.32-0.52	6.95	<0.001	10
Ferritin (ng/mL)	1.43	1.12-1.85	2.85	0.004	15	1.00	1.000-1.001	5.42	<0.001	14
Eosinophils (%)	0.63	0.45-0.85	2.79	0.005	16	0.62	0.49-0.76	4.35	<0.001	15
Hb (g/dL)	0.89	0.81-0.98	2.41	0.016	17	1.12	1.04-1.19	3.23	0.001	17
Eosinophils (10^9^/L)	0.02	0.00-0.62	2.07	0.039	18	0.04	0.003-0.360	2.67	0.007	18
Monocytes (10^9^/L)	0.72	0.45-1.07	NA	0.127	NA	0.74	0.51-1.04	1.66	0.097	20
Platelets (10^9^/L)	1.00	1.00-1.00	NA	0.203	NA	1.00	0.997-1.000	1.80	0.070	19

AST, Aspartic Amino Transferase; ALT, Alanine Amino Transferase; CRP; C Reactive Protein; GFR, Glomerular Filtration Rate; Hb, Hemoglobin; NLR, Neutrophil Lymphocyte Ratio; NA, not applicable (Z values not directly comparable), WBC, White Blood cell Count.

**Figure 5:**
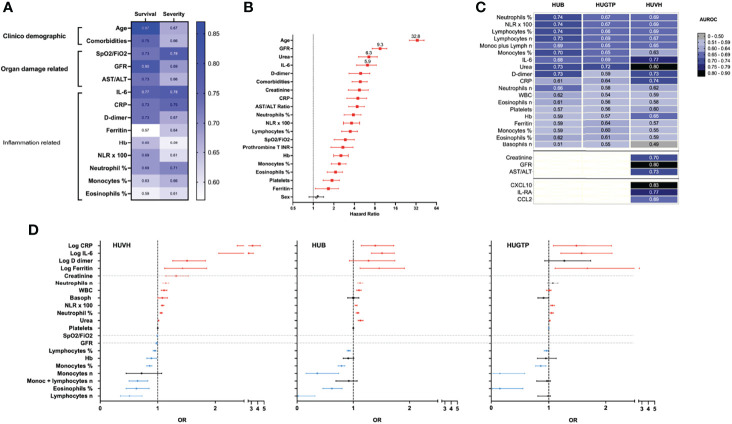
Relative weight of different variables in prediction and performance. **(A)** Heatmap summarizing the values under the curve (AUC) generated by applying Receiver Operating Characteristic (ROC) curve routinely used to assed the performance of clinical laboratory tests, to each the main variables; the performance was assessed by survival/decease and for non-severity/severity outcomes in the HUVH cohort. **(B)** Hazard ratios corresponding to survival curves for Youden index cut-off. Red, significant values for the HUVH cohort. **(C)** Heatmap of the area under the curve (AUC) of ROC curves corresponding to the variables available for the three cohorts (HUB, HGTP and HUVH). The values have grouped by unbiased hierarchical clustering. IL-6, CRP, urea, lymphocytes, and neutrophils occupy central positions. At the bottom, the AUC for some variables available only from the HUVH cohort and the AUC values for the three cytokines that perform better in the group of 74 patients who were analyzed in the HUVH cohort. The numbers within the cells are the AUC values. **(D)**. Multivariable logistic regression analysis, age-adjusted, for the main variables of the three cohorts (HUB, HGTP and HUVH). The three forest plots show how, after correcting for age, the APRs rank above the glomerular filtration rate (GFR) in the HUVH cohort and have a similar ranking in the three cohorts. The horizontal whiskers represent the 95% confidence intervals; values in red indicate positive predictive and blue negative predictive value for the 28-day survival/deceased outcome. The dotted lines indicate variables only available for HUVH. The OR rankings -differently from the ROC AUCs- are useful only to compare the different hospital cohorts, but not to compare the weight of the variables within each cohort, as ORs are derived from variables that use different units and ranges of variation. APR, acute-phase reactants. APR, acute-phase reactants; SpO2/FiO2, oxygen saturation/fraction of inspired oxygen; NLR, neutrophil-to-lymphocyte ratio; CRP, C-reactive protein; AST, aspartate aminotransferase; ALT, alanine aminotransferase; GFR, glomerular filtration rate; IL, interleukin; LDH, lactate dehydrogenase; Hb, hemoglobin; ROC, receiver-operating characteristic curve; AUC, area under the curve; HUB, Hospital Universitari Bellvitge, HUGTP, Hospital Universitari Germans Trias Pujol Hospital; HUVH, Universitari Vall Hebron.

Applying this classification to assess clinical parameters and test performance using ROC curve analysis (**Table 5**), it emerged that the CD and ODRB performed better at predicting survival, while IFRB performed better at predicting disease severity (**Figure 5A** and **Table 3**). The strong influence of age was more evident in the analysis of survival curves (**Figure 5S**) using Youden index for the cut-off values (**Table 5**); the hazard ratio (HR) for patients age under or above 60 years was 32, while the next highest HR was for GFR 9.3 (**Figure 5B**). The predictors of disease severity in descending order were age, GFR, urea, IL-6, D-dimer, and comorbidities (**Figure 5B**). The predictive power of both the ODRB and IFRB variables was maintained in the age-adjusted logistic regression analysis (**Table 4**) but reduced when ROC analysis was stratified by age intervals (**Table S8**). The random forest simulation further confirmed that age was the single best predictor of outcome, and that the combination with all laboratory variables was only partially additive (**Table S9**).

**Table 5:** ROC curve analysis as for clinical laboratory test performance comparison for survival/decease and non-severe/severe outcomes.

Decease Outcome				
Variable	AUC	CI	*p*	YOUDEN forHazard Ratios cut off
Age	0.87	0.85-0.89	0.001	>60.50
Comorbidities	0.75	0.72 -0.78	5.95E-25	>1.50
GFR	0.80	0.77-0.83	2.45E-53	< 87.30
IL-6	0.77	0.73-0.81	5.24E-26	> 50.50
AST/ALT	0.73	0.69-0.77	6.08E-31	> 1.64
SpO2/FiO2	0.73	0.70-0.78	1.79E-43	< 439.50
CRP	0.73	0.70-0.78	1.44E-20	>11.13
D-dimer	0.73	0.69-0.77	7.42E-20	> 353.00
Creatinine	0.71	0.67-0.74	4.35E-25	> 1.12
Lymphocyte %	0.69	0.65-0.73	1.18E-21	< 12.05
NLR	0.69	0.65-0.73	1.33E-21	>6.85
Neutrophil %	0.69	0.65-0.73	1.61E-43	> 82.15
Hb	0.65	0.62-0.69	5.67E-09	< 13.45
Monocytes	0.63	0.59-0.67	5.05E-11	< 6.65
Prothrombin time (INR)	0.61	0.56-0.65	1.37E-07	> 1.21
Platelets	0.60	0.56-0.64	9.94E-07	< 162.50
Eosinophils %	0.59	0.55-0.62	1.32E-5	< 0.15
Ferritin	0.57	0.51-0.62	0.019	> 668.00
**Severity outcome**				
IL6	0.78	0.75-0.80	8.65E-56	NC
SpO2/FiO2	0.77	0.74-0.81	1.79E-43	NC
CRP	0.75	0.71-0.77	5.77E-43	NC
NLR	0.71	0.68-0.73	1.74E-41	NC
GFR	0.69	0.65-0.71	1.69E-33	NC
Age	0.67	0.64-0.70	2.55E-29	NC
D-dimer	0.67	0.63-0.69	2.63E-20	NC
Monocytes %	0.66	0.62-0.68	1.92E-24	NC
AST/ALT	0.66	0.62-0.68	2.97E-24	NC
Comorbidity	0.65	0.62-0.68	5.95E-25	NC
Ferritin	0.64	0.60-0.67	1.08E-12	NC
Eosinophils %	0.61	0.57-0.63	3.14E-12	NC
Hb	0.59	0.55-0.62	5.67E-09	NC
Monocytes n	0.57	0.53-0.59	1.02E-05	NC

AST, Aspartic Amino Transferase; ALT, Alanine Amino Transferase; CRP; C Reactive Protein; GFR, Glomerular Filtration Rate; Hb, Hemoglobin; NLR, Neutrophil Lymphocyte Ratio; NC, not calculated as HR applies to mortality.

### Predictive Power of Laboratory Variables During Hospitalization

**Figure 6:**
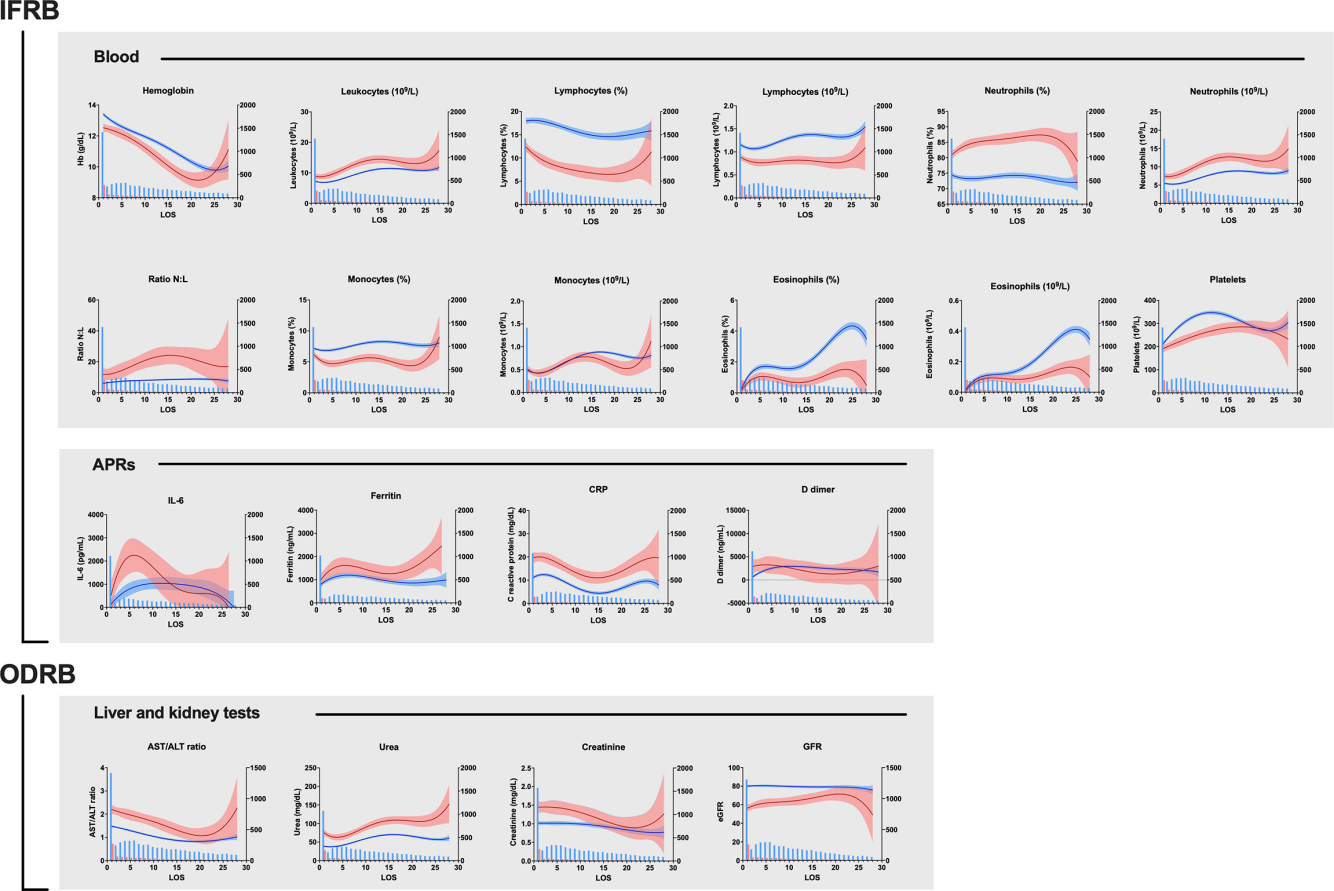
Vall d’Hebron University Hospital cohort, variations in the average clinical laboratory variables during the 28-day follow-up period. The blue and red lines represent the mean ± CI values of the parameter for each day of follow-up for the survivors and deceased respectively. The blue bars indicate the number of values available for each day. Notice that samples were not obtained every day and therefore the averages result from plotting together all available values for each day of follow-up, as in 4. Data correspond to 7,586 samples, 6,589 from survivors and 997 from deceased out of 1,079 patients of the HUVH cohort. NLR, neutrophil-to-lymphocyte ratio; CRP, C-reactive protein; AST, aspartate aminotransferase; ALT, alanine aminotransferase; GFR, glomerular filtration rate; IFRB, inflammation-related biomarkers, ODBRs, organ damage-related biomarkers. APRs, acute-phase reactants.

The analysis of the 7,586-follow-up observations showed that association of biomarkers with survival varied during the 28 days of follow-up. The daily average curves of most IFRB for survivors and deceased remained separated during the first few days of hospitalization with maximum separation around day 5 (**Figure 6**). Interpretation of the values in patients with longer hospital stays was difficult due to the decreasing sample size and complications arising from medical interventions. The survival curves for ODRBs, GFRs and AST/ALT ratio maintained their separation for most of the follow-up period.

### Selection of a Core Panel of Clinical Laboratory Tests

At present in HUVH, as in many hospitals, approximately 30 clinical laboratory variables and SpO2/FiO2 are routinely measured in COVID-19 patients as part of the work-up on admission. Correlation analysis and multivariable logistic regression showed that these variables had a high level of multicollinearity which was confirmed by PCA and random forest simulation (**Figure 3S** and **Table S9**). Using iterative logistic regression analysis with different variable combinations, a reduced set of eight variables: age, comorbidity index, SpO2/FiO2, hemoglobin, NLR, CRP, AST/ALT ratio, and GFR, were found to capture the best prediction power (see supplementary material, “Sequence of statistical biomarkers analyses: complexity reduction” and tables “Repeated multivariable logistic regression deceased” and “Repeated multivariable logistic binary severity” among the supplementary excel tables). As age and comorbidities are non-time-varying, only six of the eight variables are required for clinical management. These results apply to the cohort but do not imply that IL-6, ferritin, lactate dehydrogenase, triglycerides, procalcitonin, D-dimer, and coagulation tests do not provide valuable information in clinical practice depending on the context.

### Comparison With the Two Validation Cohorts

The comparison among the three cohorts confirmed the prognostic power of the main IFRB and ODRB variables, even though the statistical ranking of their positions showed small variations between cohorts (**Table 6**, and **Figures 5C, D**). In addition, biomarker performance as predictors of outcome was maintained in the three cohorts in the random forest simulations (**Table S10**).

**Table 6:** Pairwise comparison of demographic and clinical laboratory biomarkers in the exploratory (HUVH) and the two validation cohorts (HUGTP and HUB).

Patients	Cohort HUVH (n = 1579)	Cohort HUB (n = 598)	Cohort HUGTP (n = 423)	HUVH vs. HUB (*p*-value)	HUVH vs. HUGTP (*p*-value)	HUB vs. HUGTP (*p*-value)
**Demographics**						
Age Median (IQR)	62 (50–75)	65 (53–74)	62 (52–71)	0.07	0.94	0.03
Females n (%)	699 (44.2 %)	208 (34.8 %)	157 (37.1 %)	<0.0001	0.46	0.009
Males n (%)	880 (55.7 %)	389 (65.2 %)	266 (62.90 %)			
**Mortality**						
Global n (%)	255/1579 (16.14 %)	154/598 (25.7%)	52/423 (12.3 %)	<0.0001	<0.0001	0.57
Females n (%)	107/699 (15.3 %)	52/208 (25.0 %)	14/157 (8.9%)	0.002	0.0001	0.42
Males n (%)	148/880 (16.8%%)	101/389 (25.9 %)	38/266 (14.3)	0.0002	0.3443	0.0003
DFSO Median (IQR)	7 (4–10)	11 (8–15)	7 (4–10)	<0.0001	>0.99	<0.0001
**Laboratory variables**	**Median (IQR)**	**Median (IQR)**	**Median (IQR)**			
**Blood**						
Hb (g/dL)	13.5 (12.3–14.5)	12.8 (11.5–13.9)	13.6 (12.5–14.7)	<0.0001	0.14	<0.0001
WBC (10^9^/L)	6.6 (5.1–8.8)	7.2 (5.3–11.1)	6.9 (5.2–9.3)	<0.0001	0.07	0.07
Neutrophils, %	76.1 (68–83.2)	80.4 (68.8–87.9)	79.5 (71.2–85.4)	<0.0001	0.0001	0.33
Neutrophils (10^9^/L)	4.9 (3.5– 6.9)	5.7 (3.6–9.1)	5.2 (3.8–7.8)	<0.0001	0.005	0.17
Lymphocytes (%)	16 (10.5–23)	12.7 (7.3–20.7)	12.5 (8.1–19.1)	<0.0001	<0.0001	0.83
Lymphocytes (10^9^/L)	1 (0.7–1.4)	0.9 (0.6–1.3)	0.8 (0.6–1.2)	<0.0001	<0.0001	0.09
Monocytes (%)	6.7 (4.8–8.8)	5.8 (3.6–8.9)	6.8 (4.9–9.1)	<0.0001	0.29	<0.0001
Monocytes (10^9^/L)	0.4 (0.31–0.6)	0.4 (0.3–0.6)	0.5 (0.3–0.7)	0.34	0.009	0.003
Eosinophils (%)	0 (0–0.3)	0.1 (0–0.9)	0.1 (0–0.2)	<0.0001	0.47	0.006
Eosinophils (10^9^/L)	0 (0–0.02)	0.01 (0–0.06)	0 (0–0)	<0.0001	<0.0001	<0.0001
Basophils (10^9^/L)	0 (0.01–0.03)	0.02 (0.01–0.03)	0 (0–0)	0.06	<0.0001	<0.0001
NLR	4.8 (3.0–7.0)	6.3 (3.4–11.8)	6.3 (3.7–10.8)	<0.0001	<0.0001	0.93
Platelets (10^9^/L)	197 (154–251)	234.5 (174.3–327.8)	203 (160–254)	<0.0001	0.35	<0.0001
**Clinical Chemistry**						
CRP (mg/dL)	8.9 (3.8–16.6)	8.1 (3.5–16.7)	9.5 (4.8–16.7)	0.31	0.64	0.69
IL-6 (pg/mL)	45.1 (23.6–80.0)	53.5 (20.4–131.1)	47.1 (24.6–92.6)	0.01	0.21	0.21
Ferritin (ng/mL)	539 (282.5–1011.5)	1210.4 (617.0–1903.3)	614 (316.5–1080)	<0.0001	0.16	<0.0001
Triglycerides (mg/dL)	121 (92–161)	173.9 (123.5–251.3)	NA (NA)	<0.0001	NA	NA
LDH (UI/L)	336 (271–421)	330.1 (261.3–448.1)	313 (224–441.5)	0.50	0.02	0.04
Urea (mg/dL)	35 (25–51)	39 (26.8–58.9)	34.9 (25–48.5)	0.009	0.17	0.009
AST (IU/L)	40 (30–60)	35.4 (23.9–58)	NA (NA)	<0.0001	NA	NA
ALT (IU/L)	28 (19–50)	33.1 (19.9–60.5)	30 (21–53)	<0.0001	0.008	0.11
Direct bilirubin (mg/dL)	0.3 (0.2–0.4)	NA (NA)	NA (NA)	<0.0001	NA	NA
Total bilirubin (mg/dL)	0.6 (0.5–0.7)	0.5 (0.4–0.7)	0.6 (0.5–0.8)	<0.001	0.03	<0.0001
D dimer (ng/mL)	263 (168–463.5)	590 (355–2108)	670 (415–1188.5)	<0.0001	<0.0001	0.69
Fibrinogen (g/L)	5.1 (4.4–6)	5.8 (5.1–6.6)	7.6 (6.5–8.8)	<0.0001	<0.0001	<0.0001
Prothrombin time INR	1.1 (1.0–1.2)	1.1 (1.1–1.2)	1.22 (1.1–1.4)	<0.0001	<0.0001	<0.0001

AST, Aspartic Amino Transferase; ALT, Alanine Amino Transferase; CRP; C Reactive Protein; GFR, Glomerular Filtration Rate; Hb, Hemoglobin; NLR, Neutrophil Lymphocyte Ratio; WBC, White Blood cell Count, NA, Not Available.

### Prediction Performance of Immunological Tests

**Figure 7:**
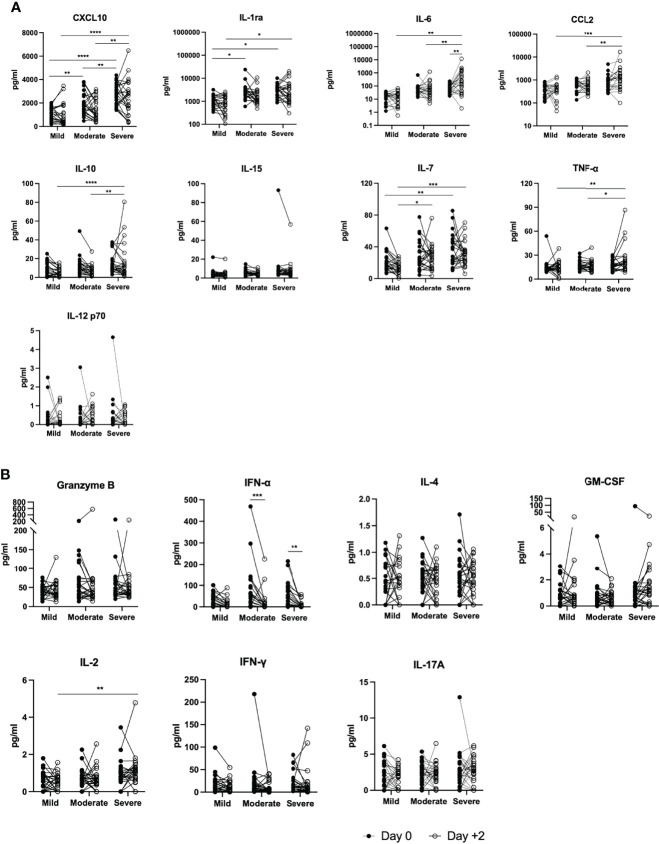
Levels of cytokines and related factors in the Vall d’Hebron University Hospital cytokine studies sub-cohort. The levels of cytokines were measured in the ELLA^®^ platform cytokines on days 0 and +2, and the changes in the levels are shown as before/after graphs. **(A)** cytokines mediating innate immunity and **(B)** Granzyme N, IFN-alpha plus cytokines mediating mostly adaptive immunity. Outliers, defined as values above mean + 2SD were identified for seven values. The three single confirmed outlier values for IL6, CXCL10 and CCL2 correspond to an early sample (day 3) of the same patient, a 44-years-old male born, in South America, without comorbidities, with severe COVID; despite the cytokine storm this patient survived and was discharged after over six weeks in hospital, four of them at the ICU with mechanical ventilation. He received two doses of TCZ as part of the treatment. Another three confirmed outlier values for IL-15, IL-12p70 and GM-CSF corresponded to early samples from a single patient, a 61-year-old female with severe pneumonia, chronic lung disease, hypertension, and obesity as risk factors; she survived and was discharged after 30 days in hospital most of them in the ICU with mechanical ventilation. A third patient, with had a single outlier value for IFN-alpha corresponded to a 61-year-old female, with diabetes and hypertension; she suffered moderate COVID, remained at the regular hospital ward and was discharged after two weeks; patients was doing well at censoring time *p < 0.05; **p < 0.01; ***p < 0.001; ****p < 0.0001.

Despite the limited size of the group analyzed in the cytokines pilot study (n=74, **Table S10**), CXCL10 had the best ROC curve (AUC=0.83, p=2.3·10^-6^) of all variables including age, IFRB and ODRB, and performed better than any of the other variables considered. IL1RA and CCL2 with AUCs of 0.77, p=0.002, and 0.69, p=0.006 respectively also showed promise as biomarkers (**Table 7** and **Figures 5C**, **7**). IL-6, also an immunological test, measured both as part of the routine clinical tests (n=1269) and in this smaller group (n=74), gave the similar AUCs of 0.77, p = 5.2·10^-26^ and 0.76, p= 1.9·10^-4^ in the two set of measurements respectively.

**Table 7:** Performance of expanded immunological parameters in the special immunological studies group as assessed by ROC curve analysis and compared with other variables in the same group.

Survival/decease as outcome											
Variables		AUC	CI	*p*-value		AUC t1	CI	*p*-value	AUC t2	CI	*p*-value
Age		0.55	0.42-0.69	0.438							
Clin Lab Biomarkers					**Cytokines**						
GFR		0.55	0.46-0.74	0.176	CXCL10	0.83	0.74-0.92	2.34E-06	0.77	0.65-0.88	1.24E-04
IL-6		0.60	0.66-0.88	**0.000**	IL-1RA	**0.77**	0.60-0.84	0.002	**0.74**	0.61-0.85	7.71E-04
SpO2/FiO2 ratio		0.77	0.70-0.91	**<0.001**	IL-6	**0.76**	0.65-0.87	1.903E-04	**0.83**	0.73-0.93	2.34E-06
CRP		0.80	0.61-0.86	**0.002**	CCL2	0.69	0.56-0.82	0.006	**0.81**	0.69-0.91	1.60E-05
D-dimer		0.73	0.43-0.71	0.353	IL-10	0.67	0.54-0.79	0.019	**0.72**	0.60-0.84	0.0014
Creatinine		0.57	0.49-0.78	0.069	IL-15	0.67	0.54-0.80	0.012	**0.74**	0.63-0.85	5.00E-04
Lymphocytes, %		0.63	0.62-0.86	**0.001**	IL-7	0.65	0.52-0.79	0.027	**0.73**	0.62-0.85	8.036E-04
NLR ×100		0.74	0.63-0.87	**0.001**	TNF-α	0.65	0.51-0.79	0.033	**0.73**	0.55-0.81	0.008
Neutrophils, %		0.75	0.66-0.89	**0.000**							
Hb		0.52	0.37-0.68	0.775							
Monocytes, %		1.00	1.00-1.00	**<0.001**							
Platelets		0.52	0.38-0.66	0.789	**Flow cytometry**						
Eosinophils, %		0.58	0.45-0.72	0.263	CD3+CD62L+ Naive T cells (%)	0.73	0.54-0.92	0.032	NA	NA	NA
Ferritin		0.61	0.46-0.76	0.147	TH17 (n)	0.67	0.45-0.88	0.131	NA	NA	NA
**Non-severe vs severe as outcome**											
**Variables**		**AUC**	**CI**	***p*-value**		**AUC t1**	**CI**	***p*-value**	**AUC t2**	**CI**	***p*-value**
Age		0.55	0.42 to 0.69	0.438							
Clin Lab Biomarkers					**Cytokines**						
GFR		**0.60**	0.45 to 0.74	0.180	CXCL10	**0.83**	0.74-0.92	2.340E-06	**0.77**	0.65-0.89	1.24E-04
IL-6		0.77	0.65 to 0.88	**2.72E-04**	IL-6	**0.77**	0.66-0.88	1.903E-04	**0.83**	0.73-0.94	2.34E-06
SpO2/FiO2 ratio		0.80	0.70 to 0.91	**3.08E-05**	IL1-RA	**0.73**	0.60-0.84	7.714E-04	**0.74**	0.62-0.86	7.71E-04
CRP		0.73	0.61 to 0.86	**0.002**	IL-15	**0.72**	0.55-0.81	0.012	**0.75**	0.63-0.86	4.98E-04
D-dimer		0.57	0.43 to 0.71	0.353	CCL-2	0.69	0.57-0.82	0.065	**0.81**	0.69-0.92	1.60E-05
Creatinine		0.63	0.49 to 0.78	0.069	IL-10	0.67	0.54-0.79	0.019	**0.73**	0.61-0.85	0.001
Lymphocytes, %		0.74	0.62 to 0.86	**0.001**	TNF-alpha	0.65	0.51-0.79	0.033	0.69	0.56-0.82	0.008
NLR ×100		0.75	0.63 to 0.87	**6.08E-04**	IL-7	0.64	0.50-0.77	0.051	**0.74**	0.62-0.85	8.04E-04
Neutrophils, %		0.77	0.66 to 0.89	**1.77E-04**	IL-2	0.6	0.46-0.73	0.176	**0.78**	0.66-0.90	7.23E-05
Hb		0.52	0.37 to 0.68	0.775	IL-17	0.52	0.38-0.66	0.777	**0.70**	0.57-0.83	0.005
Monocytes, %		0.72	0.60 to 0.84	**0.003**							
Platelets		0.52	0.38 to 0.66	0.789	**Flow cytometry**						
Eosinophils, %		0.58	0.45 to 0.72	0.263	CD3+CD62L+ Naive T (%)	0.61	0.40 to 0.83	0.3080			
Ferritin		0.61	0.46 to 0.76	0.147	TH17 (n)	0.57	0.34 to 0.79	0.5490			

Cytokine group of patients, n=74, Flowcytometry phenotype group of patients n= 41, for details see **Table S10**.

T1, time 1, initial data, T2 time 2, 2-3 days after onset. CRP; C Reactive Protein; NLR, Neutrophil/Lymphocyte Ratio; GFR, Glomerular Filtration Rate; Hb, Hemoglobin; NA, Not Available. P valued in bold, significant.

**Figure 8:**
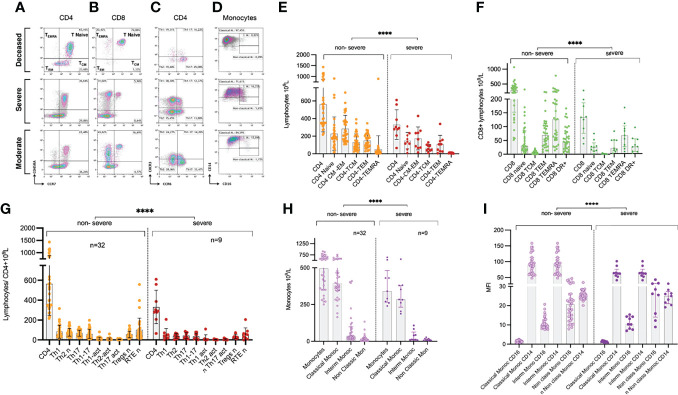
Representative flow cytometry plots from the Vall d’Hebron University Hospital sub-cohort. **(A)** CD4 and **(B)** CD8 T lymphocyte subpopulations distributed by phenotypes based on CD45RA and CCR7. **(C)** CD4 T lymphocyte Th-polarisation by CXCR3 and CCR6 expression. **(D)** Monocyte subpopulations (classical, intermediate monocytes [IM] and non-classical monocytes) in a comparison of patients belonging to the deceased, severe, and moderate patient categories. **(E)** Distribution of CD4 naïve and memory subsets among non-severe and severe patients. **(F)** Distribution of CD8 naïve and memory subsets among non-severe and severe patients; **(G)** Distribution of CD4 Th polarized subsets among non-severe and severe patients; **(H)** Distribution of monocytes subsets among non-severe and severe patients; **(I)** Mean Fluorescent Intensity (MFI) of CD14 and CD16 in the different monocyte subsets among non-severe and severe patients. Non severe patients n=32 and severe patients n=9, for all plots; ****p < 0.0001 by non-parametric FDR corrected Kruskal-Wallis test.

The immune phenotype was analyzed in 41 patients (**Table S11**). There was a steep reduction in the size of all T-cell subsets, which was more marked for CD8 effector and memory cells, and an increase in activation markers that was like the pattern observed in other time-series analyses (25, 37), revealing a deep disturbance of the immune response in severely ill patients (see Expanded phenotype analysis in supplementary). Naïve T cells seemed associated to higher mortality (**Figures 8** and **11S**).

## Discussion

The analyses of our COVID-19 patients series expose the limitations of the clinical laboratory tests currently applied to assess the prognosis of patients with COVID-19 but also show that they can be better interpreted if grouped into categories that reflect the two main biological processes that are measured, i.e., inflammation and organ damage. Since their prognostic limitations are due to redundancy, clinical laboratory panels for COVID-19 could be simplified, but additional biomarkers with real independent additional predictive power are urgently needed. This study also exposes the lack of tests for early prediction of the immune response to SARS-CoV-2. Such tests could provide critical non-redundant information required for clinical management during the early clinical course. The results of the reported pilot study using a selection of robust immunological tests in use in other areas of clinical immunology (primary immunodeficiencies, transplantation, etc.), indicates that such tests exist, and their value should be systematically investigated.

Beyond this central message, the findings can be summarized as follows: 1) The three cohorts confirmed the strong association of: SpO2/FiO2, neutrophilia, lymphopenia, acute phase reactants, coagulation factors, kidney function and the AST/ALT ratio with disease outcome. 2) There was a high level of collinearity (redundancy) among the different laboratory variables, which explains their disappointing prediction power when combined. 3) After reducing overall redundancy, the best combination of variables was age, comorbidity index, SpO2/FiO2, NLR, CRP, AST/ALT ratio, fibrinogen, and GFR. 4) The classification of biomarkers into inflammation and organ-damage related helps with their interpretation and revealed that organ damage are better predictors of survival than severity while inflammatory are better predictors of severity than of decease. 5) For the clinician at the bedside, some laboratory organ damage changes such as GFR reduction, may be less conspicuous than acute phase reactants increase but they may deserve attention when they deteriorate, even when they are still close to the normal range.

It is relevant that as part of another ongoing study lead by our institution (manuscript in preparation) antibodies to IFN-alpha2 and IFN-omega were measured in 917 patients of the HUVH cohort; 50 (5.6%) were found positive for one of the IFNs; the demographic features of these positive patients were concordant with previous reports (11, 38) in which age, mortality and male/female ratio were higher in IFN-antibody positive patients, although without significant association. Inflammation and organ damage related variables were significantly higher in the IFN-positive patients (data not shown). Differences in design preclude to incorporate these data into the current project

The study here reported is similar to a number of studies carried out during the first year of the pandemic that already detected age, sex, comorbidities and the laboratory parameters used to assess severity in sepsis, to be associated with poor prognosis in COVID-19 patients (14, 16, 17, 20, 22). Differently from many of the studies that analysed cohorts over 1000 patients, in our studies the main database was generated by the physicians attending the patients and curated by medically qualified staff this providing a reliable medium granularity data set relatively unique. The statistical analysis included a variety of techniques that eventually revealed the weaknesses of the clinical laboratory tests. The explanation became only obvious by the careful analysis of the overall correlogram, the random forest and the iterative differential logistic regression. Among organ damage associated biomarkers, the best was SpO2/FiO2, but this is a bedside test performed by health personnel that reflects impairment in gases exchange due to important lung or circulatory system damage. However, it is a late predictor of the disease severity and only appear when the damage is well stablished.

We followed 4 study in the analysis of the variation of laboratory parameters along the period of hospitalisation; in their study sTNFRSF1A, sST2 (IL-33 soluble receptor), IL-10 and IL-15 maintained separated trajectories for survivors vs deceased over the hospitalization period. In our case, lymphocyte and neutrophil percentage, the corresponding NLR, and CRP also maintained a different trajectory over almost 30 days. Of note, IL-6 showed a marked peak at day 3-5 in deceased patient that is reminiscent. but sharper, than that of sST2 curve in 4. Probably, close monitoring of these and other immunological parameters during these critical 1-5 days of hospitalisation would be a valuable tool for patient management. However, in our study, the two patients with the combined higher cytokines values survived, probably because they were relatively young (see legend to **Figure 7**). Of note, the best candidate biomarker that we detected, CXCL10 was also reported as such in 39 study,

There are several limitations to this study, starting with its retrospective nature, the relative smaller size of the mild group and the absence of non-hospitalized patients. In common to other retrospective studies, the *a priori* power calculation of the sample size was not carried out and the strategy was just to collect the maximal number of informative cases. The large cohorts obtained proved sufficient to detect the strong association of most variables considered but we cannot exclude that minor associations have been missed. The group of patients with mild COVID-19 was 71, small compared to the other groups in our study, but comparable to other retrospective studies e.g., 4. Regarding the lack of a non-hospitalised group, in fact the overall median hospital stay of the mild patients’ group was 2 days, including 46 discharged within 24h. The study here reported is being followed by a prospective study, recruitment now closed, in which we put special care in recruiting asymptomatic and symptomatic non-hospitalized cases. The initial analysis revealed that the inflammatory, cytokine and serological profiles show continuously progressive alterations throughout these categories (manuscript in preparation); this supports the notion that the group of mild patients here reported is similar to non-hospitalized patients in other series e.g., Jehi et al (16).

Another limitation is the absence of information regarding two key factors: the SARS-CoV-2 viral load and markers of the adaptive immune response. The SARS-CoV-2 detection techniques used during this period were diverse and not quantitative. This, added to the variability of swab sampling efficiency, made non-viable to include this important parameter (6) in the present study. Serological markers need 7–21 days to become detectable and are not too helpful as a tool to predict the prognosis of the patients during the initial medical assessment (39, 40). Finally, the effect of treatment on the outcomes was not analysed because therapeutic protocols and inclusion criteria were not uniform during the first wave. A summary of pharmacological treatments is however provided for reference (**Table S3**); it shows that, as in many centres in Europe and the US, hydroxychloroquine, lopinavir/ritonavir and azithromycin were administered to most patients, while corticosteroids and tocilizumab, only later confirmed to be effective, were administered to 18 to 25 percent of patients respectively with a rapid clinical deterioration; soon later these were the predominant treatments in patients requiring oxygen supplementation. We did not strictly follow the transparent reporting of a multivariable prediction model for individual prognosis or diagnosis recommendations, as generating a prediction mode was not an objective, but most requirements were fulfilled (41).

The analyses presented here are intended for improved interpretation of available tests, but no algorithm is proposed. Most algorithms with good predictive power include parameters, such as oxygen requirements and imaging data, that reflect organ damage in patients that are already on the path to severe disease; in fact they predict what was already starting to happen (14–22, 41). By contrast, the ideal test/algorithm should be able to identify patients at risk before organ damage occurs. Our results suggest that this is difficult with current tests because inflammation has limited discriminatory power and by the time organ damage biomarkers are elevated, the progress towards severity of the process is already set in motion and beyond the ideal window for an immunomodulatory intervention. If, as postulated, the main determinant of COVID-19 severity is a dysregulated innate immune response leading to an delayed adaptive immune response, immunological biomarkers of this failure should be investigated in more detail during initial infection period (42, 43). The severity of COVID19 in patients with IFN pathway genetic defects (10) or autoantibodies to type I IFNs (11) supports this notion and suggests that anti-IFN antibodies should be included in future protocols. Alternatively, being the generation of specific cytotoxic T lymphocytes the main defense mechanism against an acute infection to a novel virus, monitoring these cells or a surrogate marker of them could help to predict patient outcome (4, 23, 25, 37, 44–46). These are two obvious approaches, among many, to identify better biomarkers and the corresponding tests. This would require overcoming some technical difficulties but also the bureaucratic obstacles in transferring tests from the research to the clinical diagnostic immunology laboratories. Reliable early biomarkers would reduce the rate of hospitalisation and guide new treatment prescription to benefit patients at high-risk; generation of such biomarkers is urgent and should be feasible.

## Hospital Vall d’Hebron COVID-19 Immune Profile Group

Artur Llobell Uriel MD, Romina Dieli MD, PhD and Roger Colobran PhD, Immunology Department; Gemma Codina MD, PhD and Tomas Pumarola MD, PhD, Microbiology Department; Roser Ferrer PhD and Vicente Cortina BSc, Clinical Laboratories Department; Magda Campins MD, PhD, Epidemiology and Public Health Department; Isabel Ruiz MD, Nuria Fernaíndez MD, Esteban Ribera MD and Joan Roig MD, Infectious Diseases Department; Ricardo Ferrer MD and Adolfo Ruiz-Sanmartín MD, Intensive Care Medicine Department; Albert Selva MD, PhD and Moises Labrador MD, PhD, Internal Medicine Department; María José Soler Romeo MD PhD, Nephrology Department; Jaume Ferrer MD, PhD, Eva Polverino MD, PhD and Antonio Alvarez MD, PhD, Pneumology Department; María Queralt Gorgas PhD and Marta Miarons PhD, Clinical Pharmacy Department; Pere Soler-Palacin, MD, PhD and Andrea Martin, MD, Pediatrics Department; Anna Suy MD, Obstetrics and Gynecology Division; Maria Jose Buzón PhD and Meritxell Genescà PhD, Infectious Disease Research Group; Santiago Perez-Hoyos and Miriam Mota-Foix, Statistics and Bioinformatics Unit, Vall Hebron Research Institute (VHIR).

## Data Availability Statement

Deidentified data tables will shared on request after approval of a proposal, with a signed data access agreement. Requests to access the datasets should be directed to RP-B, Ricardo.pujol@uab.cat; MH-G, manhernandez@vhebron.net.

## Ethics Statement

This project was approved by the institutional ethics board of the three institutions (HUVH, HUGTP, and HUB) which waived the requirement for individual informed consent (protocol R(AG)242/2020). In the HUVH cohort, residual sera samples were transferred to the Vall d’Hebron University Hospital Biobank (PT17/0015/0047) part of the Carlos III Institute of Health network of biobanks (Number C.0006012). Written informed consent for participation was not required for this study in accordance with the national legislation and the institutional requirements.

## Author Contributions

AS-M, MH-G, MM-G, and RP-B: Conceived, designed the work and reviewed the final manuscript. DÁ-S carried out laboratory experiments, data analysis, figure generation, data base management and reviewed the Ms. RP-B generated the relational data base, performed the exploratory data analysis and wrote the first draft of manuscript. JP-P and IA-M collected the samples and carried out the flowcytometric blood phenotyping; PC-E performed part of the bioinformatic and statistical analysis; CZ-E, AT-S, and EM-C provided the HGTP patients data and reviewed the Ms. EP-C and JB-M provided the HUB patients data and reviewed the Ms. MR-B and FM-V provided data of internal medicine patients HUVH and reviewed the Ms; AA provided the SARS-CoV-2 data and reviewed the Ms; AS-P contribute to design the study, data collection and analysis, and reviewed the final version of the manuscript; AB-G provided the clinical laboratory data; JR provided the clinical the ICU data and reviewed the final version of the manuscript. All authors contributed to the article and approved the submitted version.

## Funding

This study was funded by Instituto de Salud Carlos III, Madrid, Spain, grants COV20/00416, Cov20/00654 and COV20/00388 to RP–B, AT-S, and JB-M respectively and co–financed by the European Regional Development Fund (ERDF). DÁ–S is recipient of a doctoral fellowship from the Vall d’Hebron Research Institute, Barcelona, Spain. AS-M was supported by a postdoctoral grant “Juan Rodés” (JR18/00022) from Instituto de Salud Carlos III through the Ministry of Economy and Competitiveness, Spain. AS-P was financially supported by the Spanish Ministerio de Ciencia e Innovacion, grant PID2019-104830RB-I00, and by the Departament d’Economia i Coneixement de la Generalitat de Catalunya, grant 2017SGR622 (GRBIO).

## Conflict of Interest

The authors declare that the research was conducted in the absence of any commercial or financial relationships that could be construed as a potential conflict of interest.

## Publisher’s Note

All claims expressed in this article are solely those of the authors and do not necessarily represent those of their affiliated organizations, or those of the publisher, the editors and the reviewers. Any product that may be evaluated in this article, or claim that may be made by its manufacturer, is not guaranteed or endorsed by the publisher.
